# Antiangiogenic therapy in breast cancer

**DOI:** 10.1007/s12254-017-0362-0

**Published:** 2017-11-06

**Authors:** Simon Peter Gampenrieder, Theresa Westphal, Richard Greil

**Affiliations:** 1 IIIrd Medical Department with Hematology and Medical Oncology, Oncologic Center, Paracelsus Medical University Salzburg, Müllner Hauptstraße 48, 5020 Salzburg, Austria; 2 Laboratory of Immunological and Molecular Cancer Research and Center for Clinical Cancer and Immunology Trials, Salzburg Cancer Research Institute, Salzburg, Austria; 3 Cancer Cluster Salzburg, Salzburg, Austria

**Keywords:** Breast cancer, Bevacizumab, Antiangiogenic therapy, Biomarker

## Abstract

Based on a strong rationale for anti-VEGF (vascular endothelial growth factor) treatment in breast cancer and promising preclinical data, great hopes have been placed on the anti-VEGF antibody bevacizumab. Clinical trials, however, reported conflicting results. In metastatic human epidermal growth factor receptor 2(HER2)-negative breast cancer, the addition of bevacizumab to standard chemotherapy improved consistently progression-free survival (PFS), however, without effect on overall survival (OS). In early breast cancer bevacizumab increased the pathologic complete response rate (pCR) after neoadjuvant therapy, but adjuvant trials did not demonstrate an effect on long-term survival. Unfortunately, despite extensive research, there is still no biomarker for bevacizumab efficacy available, making patient selection difficult. This review summarizes all phase III trials investigating efficacy and toxicity of bevacizumab in early, locally advanced and metastatic breast cancer. It recapitulates the main toxicities, gives an overview on biomarker studies and discusses the role and future aspects of antiangiogenic therapy in breast cancer.

## Introduction

There is a strong rationale for the usage of antiangiogenic therapies in early, locally advanced and metastatic breast cancer. The concentration of hypoxia-inducible factor(HIF-1)alpha, a key player in angiogenesis regulation, is higher in breast tumors than in normal breast tissue and is even higher in poorly differentiated lesions than in the corresponding type of well-differentiated lesions [1]. Furthermore, increased angiogenesis, measured by vascular endothelial growth factor (VEGF) expression or microvessel density, is an independent negative prognostic factor in early breast cancer [2–4].

The addition of the anti-VEGF antibody bevacizumab to standard therapy improved both progression-free (PFS) and overall survival (OS) in several advanced cancers (colorectal cancer, non-small cell lung cancer, ovarian cancer, cervical cancer). In metastatic breast cancer (MBC), however, bevacizumab did not show an OS benefit and increased the grade 3/4 toxicity rate. This led to a discussion about the clinical utility of this drug in this indication. In the neoadjuvant setting, several trials showed an improved pathologic complete response rate (pCR); however, adjuvant trials did not show any effect on disease-free (DFS) or OS. Other antiangiogenic drugs like sunitinib and sorafenib were investigated in advanced breast cancer as well, with consistently negative trial results [5, 6]. Therefore, this review focuses on bevacizumab and summarizes the available clinical data in early, locally advanced and metastatic breast cancer, respectively.

## Bevacizumab in metastatic breast cancer

### First-line trials

Four prospective phase III trials investigated the efficacy and tolerability of bevacizumab in combination with chemotherapy as first-line therapy in human epidermal growth factor receptor 2(HER2)-negative metastatic breast cancer (Table 1). The approval for this indication was based on the results of the E2100 trial, where the combination of bevacizumab 10 mg/kg on days 1 and 15 and paclitaxel 90 mg/m^2^ on days 1, 8 and 15 every 4 weeks showed a significant and clinically meaningful improvement in progression-free survival (PFS) compared to paclitaxel alone (11.8 months vs. 5.9 months; hazard ratio [HR] 0.60; *P* < 0.001) [7]. The subsequent phase III trials (AVADO and RIBBON-1), however, found a much less pronounced effect on PFS with other chemotherapy-backbones like docetaxel, capecitabine or anthracyclines [8, 9]. In AVADO the PFS difference was 1.9 months between docetaxel plus bevacizumab and docetaxel plus placebo [8]. In RIBBON-1 a PFS-difference of 2.9 months in the capecitabine cohort and of 1.2 months in the anthracycline/taxane cohort were reported ([9]; Table 1). The recently published MERiDiAN trial, using the same combination as in E2100, showed a similar median PFS of 11.0 months in the bevacizumab/paclitaxel arm compared to the E2100 trial. The control arm, however, performed much better with a median PFS of 8.8 months (5.6 months in E2100) resulting in a lower HR of 0.68 (*P* < 0.001; Table 1). None of these trials, nor a subsumption in a meta-analysis, showed a benefit in OS by the addition of the anti-VEGF antibody and the rate of grade 3/4 toxicity was increased [10, 11]. In HER2-positive disease bevacizumab did not even show a significant improvement in PFS when added to docetaxel and trastuzumab (AVEREL trial, Table 1; [12]).

**Table 1:** Phase III trials in locally advanced inoperable or metastatic breast cancer. The primary endpoint in all trials was PFS

Study	*n*	Line	Therapy	Median PFS						Median OS				ORR				Ref
				BEV		no BEV	HR	*P*	BEV		no BEV	HR	*P*	BEV		no BEV	*P*	
*E2100*	673	1^st^	Paclitaxel ± BEV 10 mg/kg	11.8	vs	5.9	*0.6*	*<0.001*	26.7	vs	25.2	*0.88*	0.16	36.9%	vs	21.2%	*<0.001*	[7]
*AVADO*	736	1^st^	Docetaxel + BEV 7.5 mg/kg vs. docetaxel + BEV 15 mg/kg vs. docetaxel + placebo	9 10.1	vs	8.2	*0.77* *0.67*	*0.045* *<0.001*	30.8 30.2	vs	31.9	*1.05* *1.03*	0.72 0.85	55% 64%	vs	46%	*0.07* *<0.001*	[8]
*RIBBON-1*	615	1^st^	Capecitabine ± BEV 15 mg/kg	8.6	vs	5.7	*0.69*	*<0.001*	29	vs	21	*0.85*	0.27	35.4%	vs	23.6%	*0.01*	[9]
*RIBBON-1*	622	1^st^	Anthracycline or taxane ± BEV 15 mg/kg	9.2	vs	8.0	*0.64*	*<0.001*	25.2	vs	23.8	*1.03*	0.83	51.3%	vs	37.9%	*0.05*	[9]
*AVEREL* ^b^	424	1^st^	Docetaxel + trastuzumab ± BEV 15 mg/kg	16.5	vs	13.7	*0.82*	*0.078*	>38	vs	>38	*1.01*	0.95	74%	vs	70%	0.349	[12]
*MERiDiAN*	481	1^st^	Paclitaxel ± BEV 10 mg/kg	11.0	vs	8.8	*0.68*	*0.0007*	NR					54%	vs	33.2%	*<0.001*	[35]
*AVG2119g*	462	2^nd^–3^rd^	Capecitabine ± BEV 15 mg/kg	4.8	vs	4.2	*0.98*	*0.857*	15.1	vs	14.5	*NR*	NR	19.8%	vs	9.1%	*0.001*	[49]
*RIBBON-2*	684	2^nd^	Taxane or gemcitabine or capecitabine or vinorelbine ± BEV 10 or 15 mg/kg	7.2	vs	5.1	*0.78*	*0.007*	18.0	vs	16.4	*0.9*	0.372	39.5%	vs	29.6%	0.193	[18]
*TANIA*	494	2^nd^ (and-3^rd^)	Taxane, capecitabine, anthracycline, gemcitabine ± BEV 10 or 15 mg/kg	6.3	vs	4.2	*0.75*	*0.007*	19.7	vs	18.7	*0.93*	0.725	21%	vs	17%	0.35	[19, 20]
*CALGB 40503* ^a^	343	1^st^	Letrozole ± BEV 15 mg/kg	20.2	vs	15.6	*0.75*	*0.016*	47.2	vs	43.9	*0.87*	0.188	69%	vs	49%	*0.04*	[16]
*LEA* ^a^	374	1^st^	Letrozole or fulvestrant ± BEV 15 mg/kg	19.3	vs	14.4	*0.83*	*0.126*	52.1	vs	51.8	*0.87*	0.518	41%	vs	22%	*<0.001*	[17]

^a^In the LEA trial and CALGB 40503 trial no chemotherapy but endocrine therapy was investigated

^b^The AVEREL trial included patients with HER2-positive breast cancer only

*PFS* progression free survival, *OS* overall survival, *ORR* overall response rate, *Ref* reference, *BEV* bevacizumab, *HR* hazard ratio, *p* p-value

These results led in November 2011 to the decision of the Food and Drug Administration (FDA) to withdraw the accelerated approval for bevacizumab for the indication breast cancer. In contrast, based on the consistently improved PFS and overall response rate (ORR), bevacizumab is still approved in Europe by the EMA as first-line therapy in HER2-negative metastatic breast cancer when combined with paclitaxel or capecitabine. However, also in European countries like Austria, a significant decline in bevacizumab prescriptions for MBC became evident after the FDA decision [13].

The two approved chemotherapy backbones, each in combination with bevacizumab, were compared within the TURANDOT trial [14]. The primary objective of this study was to show non-inferiority of capecitabine/bevacizumab when compared to paclitaxel/bevacizumab in terms of OS. The primary endpoint, in fact, was met (median OS 30.2 months vs. 26.1 months; stratified HR 1.02; *P* = 0.007 indicating non-inferiority); however, this was not supported by the unstratified analysis. In addition, the median PFS was significantly longer in the paclitaxel arm (10.9 months vs. 8.1 months 95% CI 10.8–12.9; stratified HR 1.32, 95%; *P* = 0.007) and the objective response was also significantly superior with paclitaxel compared to capecitabine (44% vs 27%; *P* < 0.001; [14]). In the ATHENA registry, including 2251 patients with HER2-negative breast cancer, the superiority of paclitaxel over capecitabine to improve time-to-progression (TTP: 9.8 months vs. 7.0 months) and response rate (49% vs. 36%) was confirmed (not statistically tested) [15].

Two phase III trials investigated the efficacy and tolerability of bevacizumab in combination with endocrine therapy for locally advanced or metastatic breast cancer. Both trials showed conflicting results: while the CALGB 40503 trial reported a statistically significant improvement in PFS by the addition of bevacizumab to letrozole (20.2 months vs. 15.6 months; HR 0.75; *P* = 0.016) [16], the LEA trial failed to show superiority (median PFS 19.3 months vs. 14.4 months; HR 0.83, *P* = 0.126) [17]. In both trials, similar benefits were observed with respect to ORR and clinical benefit rate (CBR), but again neither study showed a difference in OS.

### Second-line trials

Two phase III trials investigated bevacizumab after one prior chemotherapy-line for metastatic disease (RIBBON-2 and TANIA). Both trials showed an improvement in PFS by the addition of bevacizumab to different chemotherapy backbones (ΔPFS 2.1 months in both trials; [18, 19]). Noteworthy, all patients included in the TANIA trial, where already pretreated with bevacizumab in the first-line setting and received bevacizumab for two additional therapy lines. Thus, the study investigated the principle of treatment-beyond-progression, as it is established in HER2-positive disease with trastuzumab. The primary endpoint, second-line PFS, was significantly longer when bevacizumab was continued (6.3 months vs. 4.2 months, HR 0.75; *P* = 0.007; [19]). The secondary endpoints, third-line PFS, second- plus third-line PFS and OS, however, where not improved ([20]; Table 1). The only chemotherapy-based phase III trial in HER2-negative MBC which did not show a significant improvement in PFS was the AVG2119g trial. In this pioneer trial, published already in 2005, patients pretreated with one or two chemotherapy lines for metastatic disease where randomized between bevacizumab plus capecitabine or capecitabine alone. Both the TANIA and the AVG2119g trial indicate that an early application of the anti-VEGF antibody is essential to gain the maximal benefit. Similar to the first-line trials, no trial beyond first-line showed an improvement in OS.

### Maintenance therapy

One prospective trial addressed the question of maintenance therapy with bevacizumab after an induction phase with chemotherapy plus bevacizumab. Patients included in the IMELDA trial were treated with six cycles of docetaxel plus bevacizumab and were then randomized to maintenance therapy with bevacizumab alone or in combination with capecitabine. The combination therapy showed significantly improvement in PFS (median 11.9 months vs. 4.3 months; stratified HR 0.38; 95%CI 0.27–0.55; *P* < 0.0001; [21]) indicating that maintenance bevacizumab alone is not an effective treatment option. Furthermore, the median duration of docetaxel induction was short (3.5 months) which may have contributed to the positive effect of early second-line chemotherapy with capecitabine.

Several retrospective case series investigated the question of maintenance therapy as well. A multicenter retrospective observational study, including 314 patients with HER2-negative disease, reported a significantly longer median PFS (18 months vs. 13 months; *P* < 0.001) and OS (55 months vs. 38 months; *P* < 0.001) in patients where maintenance therapy was prescribed [22]. These results, however, must be interpreted with caution because such retrospective non-randomized trials harbor the risk of multiple biases.

## Bevacizumab in early and locally advanced operable breast cancer

### Neoadjuvant trials

Based on the clear effect on response rate in the palliative setting, three phase III neoadjuvant trials with bevacizumab were started. All three trials showed a significantly higher rate of pathologic complete response (pCR) when bevacizumab was added to neoadjuvant chemotherapy (Table 2; [23–28]). The effect on pCR, however, was modest at best (ΔpCR 5–11%) and no significant differences in DFS where reported. In the ARTEMIS trial, patients achieving a pCR in the bevacizumab group showed no longer DFS suggesting a lack of activity against micrometastases. Interestingly, the NSABP B‑40 study, which additionally used bevacizumab postoperatively, showed a statistically significant difference in OS in favor of bevacizumab (HR 0.65; 95%CI 0.49–0.88; *P* = 0.004) [25]. In the context of the other neoadjuvant and adjuvant trials showing no effect on long-term survival; this observation should not be over-interpreted.

**Table 2:** Neoadjuvant phase II/III trials in early or locally advanced breast cancer. The primary endpoint in all trials was pCR

Study	*n*	Phase	Subtype	Therapy	pCR rate			3-year DFS				3-year OS				Ref
					BEV (%)	No BEV (%)	*P*	BEV	No BEV	HR	*P*	BEV	No BEV	HR	*P*	
*GeparQuinto*	678	III	TNBC	EC → T ± BEV 15 mg/kg	39	28	*0.004*	80.8%	80.8%	*1.03*	0.784	89.7%	89.7%	*0.974*	0.842	[23, 24]
*NSABP B40*	1206	III	HER2−	TX → AC or T + gemcitabine → AC ± BEV 15 mg/kg neoadjuvant and adjuvant	35	28	*0.02*	NR	NR	*0.8*	0.06	NR	NR	*0.65*	*0.004*	[25, 26]
*ARTemis*	800	III	HER2−	T → FEC ± BEV 15 mg/kg	22	17	*0.03*	74%	78%	*1.18*	0.25	81%	85%	*1.26*	0.19	[27, 28]

*BEV* bevacizumab, *EC* epirubicine plus cyclophosphamide, *T* docetaxel, *TX* docetaxel plus capecitabine, *AC* doxorubicine plus cyclophosphamide, *FEC* fluorouracil plus epirubicine plus cyclophosphamide, *n* number of patients, *DFS* desease free survival, *HR* hazard ratio, *OS* overall survival, *pCR* pathologic complete response, *TNBC* triple negative breast cancer, *HER2* human epidermal growth factor receptor, *NR* not reached, *NG* not given, *p* p-value

A special situation for neoadjuvant treatment is inflammatory breast cancer. Since this subtype is highly angiogenic showing high microvessel density and VEGF expression, an especially strong effect of bevacizumab was expected. A recent single arm phase II trial investigated FEC followed by docetaxel both in combination with bevacizumab every 3 weeks. Postoperatively, all patients received adjuvant bevacizumab (plus endocrine therapy in case of hormone-receptor positivity). The trial did not meet the prespecified criteria for efficacy with a pCR rate of 19% and a 3-year DFS of 57% (95%CI 47–66%) and a median DFS of 53 months (95%CI 31–not estimable); however, longer follow-up is needed for definitive conclusions [29].

### Adjuvant trials

In the adjuvant setting three phase III trials were published until now (Table 3). The BEATRICE trial included patients with triple-negative disease [30, 31], the E5103 patients with HER2-negative [32] and the NSABP B44 patients with HER2-positive breast cancer [33]. The Kaplan–Meier curves for disease-free survival (DFS) and OS in all three were completely overlapping. This treatment failure can be explained by the fact that anti-angiogenic drugs can only work at a time point where neoangiogenesis is actually running, which is not the case in the adjuvant setting. Since senescent disease cannot be affected by anti-angiogenic treatment and since no clinicopathological characteristics or biomarkers have been identified to indicate bevacizumab efficacy, anti-VEGF therapy has no application in the adjuvant setting.

**Table 3:** Adjuvant phase III trials in early or locally advanced breast cancer

Study	*n*	Subtype	Chemotherapy	Primary Endpoint	DFS				OS				Ref
					BEV	No BEV	HR	*P*	BEV	No BEV	HR	*P*	
*BEATRICE*	2591	TNBC	Anthracycline and/or taxane ± BEV 5 mg/kg/week	PFS	5y 80%	5y 77%	*0.87*	0.18	5y 88%	5y 88%	*0.93*	0.52	[30, 31]
*E5103*	4994	HER2−	AC → paclitaxel ± BEV 15 mg/kg ± BEV-Maintenance	PFS	5y 80%	5y 77%	*0.87*	0.17	NR	NR	*0.89*	0.41	[32]
*NSABP B40*	1206	HER2−	TX → AC or T + gemcitabine → AC ± BEV 15 mg/kg neoadjuvant and adjuvant	pCR	NR	NR	*0.8*	0.06	NG	NG	*0.65*	*0.004*	[25, 26]
NSABP B44 BETH*	3509	HER2+	C + T + H ± BEV → H ± BEV T + H ± BEV → FEC → H ± BEV 15 mg/kg	PFS	3y 92%	3y 92%	*0.99*	0.96	3y 97%	3y 96%	*0.87*	0.44	[33]

*BEV* Bevacizumab, *AC* Doxorubicine cyclophosphamide, *TX* Docetaxel plus capecitabine, *T* Docetaxel, *C* Carboplatin, *H* Trastuzumab, *FEC* Fluorouracil plus epirubicine plus cyclophosphamide, *n* number of patients, *DFS* desease free survival, *PFS* progression free survival, *HR* hazard ratio, *OS* overall survival, *pCR* pathologic complete response, *TNBC* triple negative breast cancer, *HER2* human epidermal growth factor receptor, *NR* not reached, *NG* not given, *p* p-value

## Toxicity

In general, bevacizumab is a well-tolerated drug. The most frequent side effects when given as monotherapy are hypertension (15% grade 1/2, 5% grade 3) and proteinuria (10% grade 1/2, 2% grade 3), which are usually asymptomatic and manageable [31]. Infrequent but severe toxicities are left ventricular dysfunction (~1%), arterial thromboembolism including stroke or myocardial infarction (<1%), major bleeding (~1%), wound-healing complications (~1%), osteonecrosis of the jaw (<1%), gastrointestinal perforation or fistula (<1%), reversible posterior leukoencephalopathy syndrome (RPLS; <0.5%), and infusion reactions (<1%; [10, 15, 31]). When added to chemotherapy, the incidences of neutropenia and febrile neutropenia are slightly increased (Table 4; [10]). These side effects, however, strongly depend on the type of chemotherapy backbone: while febrile neutropenia was low with weekly paclitaxel (0.8% vs. 0%), the percentage was clearly higher with 3‑weekly docetaxel (16% vs. 11%) [7, 8]. The same applies to sensory neuropathy with grade 3/4 neuropathy in 24% vs. 18% of patients treated with paclitaxel and 3% vs. 0.5% of patients receiving capecitabine plus bevacizumab. The frequency of venous thrombotic events is not increased by the addition of bevacizumab to chemotherapy in patients with different metastatic tumors [34].

**Table 4:** Summary of selected grade ≥3 adverse events of bevacizumab in combination with chemotherapy in three phase III trials in first-line [10]. Adverse events with a >2% higher incidence in the bevacizumab group compared with the non-bevacizumab group are highlighted in italic

Grade 3/4 adverse event	BEV (%) (*n* = 1679)	No BEV (%) (*n* = 982)
*Neutropenia*	*10.0*	*7.1*
Sensory neuropathy	9.5	8.5
*Hypertension*	*9.0*	*1.2*
*Febrile neutropenia*	*6.5* ^a^	*3.5*
Venous thromboembolic event	2.8	3.8
*Proteinuria*	*2.3*	*0.0*
Arterial thromboembolic event	1.6	0.3
Bleeding	1.5	0.4
Left ventricular systolic function	1.5	0.2
Wound dehiscence	0.8	0.3
Fistula	0.5	0.3
GI perforation	0.5	0.3
RPLS	<0.1	0.0

*BEV* bevacizumab, *GI* gastrointestinal, *RPLS* reversible posterior leukoencephalopathy syndrome, *TNBC* triple-negative breast cancer

^a^ 0.8% with weekly paclitaxel in E2100 [7]

## Biomarker research

The trials results, both in advanced and in early breast cancer, call for biomarkers allowing identification of patients with or without a relevant chance of clinical benefit from this drug. Unfortunately, clinicopathologic parameters like triple-negativity or high-risk features (visceral disease, ≥3 metastatic sites, prior [neo]adjuvant chemotherapy) do not help to identify patients with special benefit from the addition of bevacizumab [10]. In a meta-analysis of the three first-line trials in metastatic breast cancer (E2100, AVADO, RIBBON-1), the hazard ratio (HR) for PFS in these subgroups was similar to the HR in the overall population ranging from 0.60 to 0.64 [10].

Several promising biomarkers like plasma levels of VEGF-A or VEGFR-2 [31, 35, 36], tissue markers like the VEGFR co-receptor neuropilin-1 (NRP-1) [37–39], single nucleotide polymorphisms (SNPs) in VEGF-A [40] or clinical markers like treatment-induced hypertension [40–42] showed convincing results in their early development as treatment predictors. However, all these markers either lack validating studies or failed to demonstrate clinical utility or reproducibility. The best example is plasma vascular endothelial growth factor-A (pVEGF-A), which showed a clear predictive value in the exploratory biomarker program of AVADO [36]. Therefore, the double-blind placebo-controlled randomized biomarker phase III trial MERiDiAN used the short isoforms of pVEGF-A as stratification factor [35]. Both co-primary endpoints of the trial were met: in the intent-to-treat (ITT) population, the stratified PFS HR was 0.68 (99%CI 0.51–0.91; *P* < 0.001) and in the VEGF-A_high_ subgroup, the stratified PFS HR was 0.64 (96%CI 0.47–0.88; *P* = 0.004). The PFS benefit, however, was similar in the pVEGF-A_low_ subgroup (HR 0.73; 95%CI 0.52–1.03). The VEGF-A-by-treatment interaction test (*P* = 0.462) for PFS in the ITT population did not support a predictive effect of pVEGF-A [35].

## Future aspects

Despite extensive research, an applicable biomarker for bevacizumab efficacy is still lacking. Further research is ongoing; however, the patent expiry of Avastin® (Roche, Basel, Switzerland) in 2018 will probably hinder further development. For the same reason, no new phase III trials in breast cancer investigating solely the addition of bevacizumab to standard therapy are expected. However, besides its antiangiogenic activity, bevacizumab has a substantial immunomodulatory capacity. VEGF-A has been shown to suppress dendritic-cell maturation, to inhibit proliferation of regulatory T cells and to attract myeloid-derived suppressor cells [43–45]. These immunosuppressive effects are counteracted by bevacizumab. It has been shown that the tumor infiltration by CD4+ and CD8+ T cells and dendritic cells was increased after bevacizumab treatment and that the antigen-presenting capacity of dendritic cells was augmented [46–48]. These effects increase the immunogenicity of tumors and could therefore increase the number of patients benefiting from immunotherapy. Because of this potential synergistic effect, several combination studies with checkpoint inhibitors and bevacizumab are ongoing in different cancer types (colorectal cancer—NCT02982694, renal cell carcinoma—NCT02724878 and NCT01984242, melanoma—NCT03175432, cervical cancer—NCT02921269, and non-small cell lung cancer—NCT02366143). In case of positivity, the immunological effect of antiangiogenic therapeutics, which has been ignored for a long time, could lead to new treatment indications and new therapeutic goals, probably also in breast cancer.

### Take home message

Bevacizumab consistently prolonged progression-free survival and increased response rate in metastatic HER2-negative breast cancer in first- and second-line. An effect on overall survival, however, was not observed stirring up a debate about the value of bevacizumab in this indication. In the neoadjuvant setting the pathologic complete response rate (pCR) was increased, however in adjuvant trials no effect on disease-free survival was reported. Therefore, antiangiogenic treatment is not standard in early and locally advanced breast cancer at least until a biomarker for patient selection is available.
